# Supplementary material for: Hot spot prediction in protein-protein interactions by an ensemble system
Source: BMC Syst Biol. 2018 Dec 31;12(Suppl 9):132. doi: 10.1186/s12918-018-0665-8 (PMC6311905; doi:10.1186/s12918-018-0665-8)
Supplement: Supplementary file 2 — All relevant MATLAB code. A simple Matlab implement of our predictor. (ZIP 19,742 kb) [file 12918_2018_665_MOESM2_ESM.zip › Additional file 2/Read ASA/ASA website/dbMPIKT/1YCRB.html]

 NetSurfP - Protein Surface Accessibility and Secondary Structure Predictions

  

|  |  |  |
| --- | --- | --- |
|  |  | NetSurfP - Protein Surface Accessibility and Secondary Structure Predictions  Technical University of Denmark |

  


---

```
# For publication of results, please cite:
# A generic method for assignment of reliability scores applied to solvent accessibility predictions.
# Bent Petersen, Thomas Nordahl Petersen, Pernille Andersen, Morten Nielsen and Claus Lundegaard
# BMC Structural Biology 2009, 9:51 doi:10.1186/1472-6807-9-51
#
# Column 1: Class assignment - B for buried or E for Exposed - Threshold: 25% exposure, but not based on RSA
# Column 2: Amino acid
# Column 3: Sequence name
# Column 4: Amino acid number
# Column 5: Relative Surface Accessibility - RSA
# Column 6: Absolute Surface Accessibility
# Column 7: Z-fit score for RSA prediction
# Column 8: Probability for Alpha-Helix
# Column 9: Probability for Beta-strand
# Column 10: Probability for Coil
E S  1YCRB                  1    0.911 106.734   0.369   0.003   0.003   0.994
E Q  1YCRB                  2    0.647 115.554  -0.328   0.430   0.016   0.555
E E  1YCRB                  3    0.668 116.769   0.059   0.622   0.015   0.363
E T  1YCRB                  4    0.370  51.291   0.362   0.694   0.003   0.303
B F  1YCRB                  5    0.092  18.545  -0.513   0.858   0.002   0.139
E S  1YCRB                  6    0.468  54.791   0.173   0.879   0.010   0.111
E D  1YCRB                  7    0.500  71.992   0.240   0.879   0.010   0.111
B L  1YCRB                  8    0.089  16.387  -0.117   0.879   0.010   0.111
B W  1YCRB                  9    0.134  32.179   0.253   0.879   0.010   0.111
E K  1YCRB                 10    0.536 110.173   1.463   0.802   0.014   0.185
B L  1YCRB                 11    0.215  39.385  -0.162   0.622   0.015   0.363
B L  1YCRB                 12    0.150  27.502   0.558   0.339   0.016   0.645
E P  1YCRB                 13    0.466  66.069  -0.499   0.115   0.016   0.868
E E  1YCRB                 14    0.766 133.768   0.129   0.053   0.005   0.942
E N  1YCRB                 15    0.921 134.864  -0.880   0.003   0.003   0.994

Explain the output.  Go back.
```

---
